# New species of oribatid mites of the genera *Lepidozetes* and *Scutozetes* (Acari, Oribatida, Tegoribatidae) from Nepal

**DOI:** 10.3897/zookeys.339.6199

**Published:** 2013-10-03

**Authors:** Sergey G. Ermilov, Jochen Martens, Andrei V. Tolstikov

**Affiliations:** 1 Tyumen State University, Tyumen, Russia; 2 Johannes Gutenberg Universität, Mainz, Germany

**Keywords:** Oribatida, new species, description, *Lepidozetes*, *Scutozetes*, key, Nepal

## Abstract

Two new species of oribatid mites, *Lepidozetes acutirostrum* **sp. n.** and *Scutozetes clavatosensillus* **sp. n.**, are described from Nepal. The genera *Lepidozetes* and *Scutozetes* are recorded for the first time for the Oriental region. The identification keys to the known species of these genera are provided.

## Introduction

In the course of taxonomic identification of Nepalese oribatid mites^1^ (Acari: Oribatida) we found two new species of the family Tegoribatidae, belonging to the genera *Lepidozetes* Berlese, 1910 and *Scutozetes* Hammer, 1952. The purpose of this paper is to describe and illustrate these species under the names *Lepidozetes acutirostrum* sp. n. and *Scutozetes clavatosensillus* sp. n.

*Lepidozetes* is a small genus that was proposed by Berlese (1910) with *Lepidozetes singularis* Berlese, 1910 as the type species. Currently, the genus comprises four^2^ species, which distributed in the Holarctic region (Subías 2004, online version 2013). Hence, the genus *Lepidozetes* is recorded in the Oriental region for the first time. The main generic characters of the genus were summarized by Bayartogtokh and Aoki (1999) and Weigmann (2006).

*Scutozetes* is a small genus that was proposed by Hammer (1952) with *Scutozetes lanceolatus* Hammer, 1952 as the type species. Currently, the genus comprises two species, which distributed in the Holarctic and Neotropical regions (Subías 2004, online version 2013). Hence, the genus *Scutozetes* is recorded in the Oriental region for the first time. The main generic characters of the genus were presented by Hammer (1952) and summarized by Bayartogtokh and Aoki (1999).

The identification keys to the known species of *Lepidozetes* and *Scutozetes* are provided below.

## Material and methods

Specimens of *Lepidozetes acutirostrum* sp. n. (holotype: female; six paratypes: four females, two males) and *Scutozetes clavatosensillus* sp. n. (holotype: female; five paratypes: three females, two males) were collected by J. Martens and A. Ausobsky from Nepal: Mustang District, Purano Marpha above the village of Marpha, eastern Dhaulagiri massif, 3200–3600 m a.s.l., forest (prevailed *Pinus wallichiana*, *Cupressus torulosa*, *Abies spectabilis*) slightly north of the Himalayan main range, soil litter, 22.IV.1980.

All specimens were studied in lactic acid, mounted in temporary cavity slides for the duration of the study, and then stored in 70% ethanol in vials. Body measurements are presented in micrometers. The body length was measured in lateral view, from the tip of the rostrum to the posterior edge of the ventral plate. Notogastral width refers to the maximum width in dorsal aspect. Lengths of body setae were measured in lateral aspect. Formulae for leg setation are given in parentheses according to the sequence of trochanter–femur–genu–tibia–tarsus (famulus included). Formulae for leg solenidia are given in square brackets according to the sequence of genu–tibia–tarsus. Terminology used in this paper mostly follows that of Norton and Behan-Pelletier (2009).

## Descriptions of new species

### Lepidozetes acutirostrum

Ermilov, Martens & Tolstikov sp. n.

http://zoobank.org/A2C12F54-D6D3-4624-9C5F-B944E4C1F09F

http://species-id.net/wiki/Lepidozetes_acutirostrum

Figs 1 –16

#### Diagnosis.

Body size 647–697 × 431–481. Body surface microfoveolate. Rostrum pointed. Anterior margin of lamellae concave medially. Interlamellar setae longer than rostral and lamellar setae. Sensilli with lanceolate head. Tutoria with one strong tooth. Notogastral setae of medium size, weakly thickened, barbed. Pedotecta I pointed anteriorly. Adanal setae *ad*_1_, *ad*_2_ longer than other anogenital setae.

#### Description.

*Measurements*. Body length 697 (holotype: female), 647–697 (six paratypes: four females and two males); body width 481 (holotype), 431–481 (six paratypes).

*Integument*. Body color brown. Body surface distinctly microfoveolate; foveolae rounded (diameter up to 1) or elongated. Dorsal sides of lamellae with longitudinal striae.

*Prodorsum*. Rostrum pointed (*t*). Lamellae long and broad, covering the prodorsum completely, except rostrum and parts of pedotecta I (Pd I). Anterior margin of lamellae concave medially. Rostral setae (*ro*, 94–106) setiform, ciliate, directed anterio-mediad, inserted laterally on prodorsum. Lamellar setae (*le*, 77–86) thickened, straight, barbed, directed forward, inserted dorso-anteriorly on lamellae. Interlamellar setae (*in*, 131–130) setiform, barbed, directed upwards and forwards, inserted on posterior part of lamellae; basal parts of these setae covered by the anterior margin of notogaster. Sensilli (*ss*, 82–94) with long stalk and elongate, lanceolate, barbed head. Tutoria (*tu*) long, of medium width, with one strong tooth anteriorly. Exobothridial setae (*ex*, 32–94) setiform, thin, slightly barbed, inserted posteriorly to tutoria.

*Notogaster*. Pteromorphs broadly rounded laterally. Anterior margins of pteromorphs with pointed tooth (*ptt*). Dorsophragmata (*D*) located close to each other. Postero-median part of hinges (*hi*) distinct, anterior part unvisible. Lenticulus (*len*) present, triangular, with amorphic borders. Four pairs of porose areas rounded: *Aa* (16–20), *A1* (12–16), *A2* and *A3* (both pairs, 8–12). Ten pairs of notogastral setae weakly thickened, barbed: posterior setae *p*_1_, *p*_2_, *p*_3_ (32–41) shorter than other setae (53–61). Lyrifissures *ia*, *im*, *ip*, *ih* and *ips* and opisthonotal gland openings (*gla*) located typically for the genus.

*Gnathosoma*. Subcapitulum longer than wide (151 × 110). Subcapitular setae *h* (28–32) thickened, straight, barbed; *a* (18–21) and *m* (41–45) thinner, slightly barbed. Two pairs of adoral setae (*or*_1_, *or*_2_, 12–14) setiform, hook-like distally, barbed. Palps (length 90–94) with setation 0–2–1–3–9(+ω). Solenidion weakly thickened, straight, blunt-ended, attached with eupathidium (*acm*). Chelicerae (length 151) with two barbed setae; *cha* (45–49) longer than *chb* (28–32). Trägårdh’s organ (Tg) long, conical.

*Lateral podosomal and epimeral regions*. Genal teeth (*gt*) narrowly triangular. Pedotecta I large, pointed (*pt*) anteriorly. Pedotecta II (Pd II) small, scale-like. Apodemes 1, 2, 3 and sejugal distinctly developed, not fused medially. Epimeral setal formula 2–1–3–3; setae (16–24) setiform, slightly barbed. Setae *1c* and their alveoli absent. Custodia (*cus*) with thin, free, blunted tips, directed anteriorly to the pedotecta II. Discidia (*dis*) pointed. Circumpedal carinae (*cp*) distinct.

*Anogenital region*. Six pairs of genital (*g*_1_–*g*_6_, 20), one pair of aggenital (*ag*, 20), two pairs of anal (*an*_1_, *an*_2_, 24) and three pairs of adanal (*ad*_1_, *ad*_2_, 36–41; *ad*_3_, 28–32) setae setiform, barbed. Lyrifissures *iad* located in paraanal position. Ovipositor elongate, narrow (192 × 61); length of lobes 86, length of cylindrical distal part 106. Lobes with 12 thin, smooth setae: ψ_1_ ≈ τ_1_ (36) longer thanψ_2_ ≈ τ_a_ ≈ τ_b_ ≈τ_c_ (16). Coronal setae *k* simple, short (8).

*Legs*. Medial claw thicker than two lateral claws; all smooth. Generally, morphology of leg segments, setae and solenidia typical for the genus (Bayartogtokh and Aoki 1999). Formulae of leg setation and solenidia: I (1–5–3–4–20) [1–2–2], II (1–5–3–4–15) [1–1–2], III (2–2–1–3–15) [1–1–0], IV (1–2–2–3–12) [0–1–0]; homology of setae and solenidia indicated in Table 1. Femora III with two setae. Famulus (*e*) short, straight, weakly blunt-ended, inserted between solenidia.

**Table 1.** Leg setation and solenidia of adult *Lepidozetes acutirostrum* sp. n. (same data for *Scutozetes clavatosensillus* sp. n.).

**Leg**	**Trochanter**	**Femur**	**Genu**	**Tibia**	**Tarsus**
I	*v*’	*d*, (*l*), *v*’’, *bv*’’	(*l*), *v*’, σ	(*l*), (*v*), φ_1_, φ_2_	(*ft*), (*tc*), (*it*), (*p*), (*u*), (*a*), *s*, (*pv*), *v*’, (*pl*), *l*’’, *e*, ω_1_, ω_2_
II	*v*’	*d*, (*l*), *v*’’, *bv*’’	(*l*), *v*’, σ	(*l*), (*v*), φ	(*ft*), (*tc*), (*it*), (*p*), (*u*), (*a*), *s*, (*pv*), ω_1_, ω_2_
III	*l*’, *v*’	*d*, *ev*’	*l*’, σ	*l*’, (*v*), φ	(*ft*), (*tc*), (*it*), (*p*), (*u*), (*a*), *s*, (*pv*)
IV	*v*’	*d*, *ev*’	*d*, *l*’	*l*’, (*v*), φ	*ft*’’, (*tc*), (*p*), (*u*), (*a*), *s*, (*pv*)

Roman letters refer to normal setae (*e* to famulus), Greek letters to solenidia. Single prime (’) marks setae on anterior and double prime (’’) setae on posterior side of the given leg segment. Parentheses refer to a pseudosymmetrical pair of setae.

**Figures 1–6.**
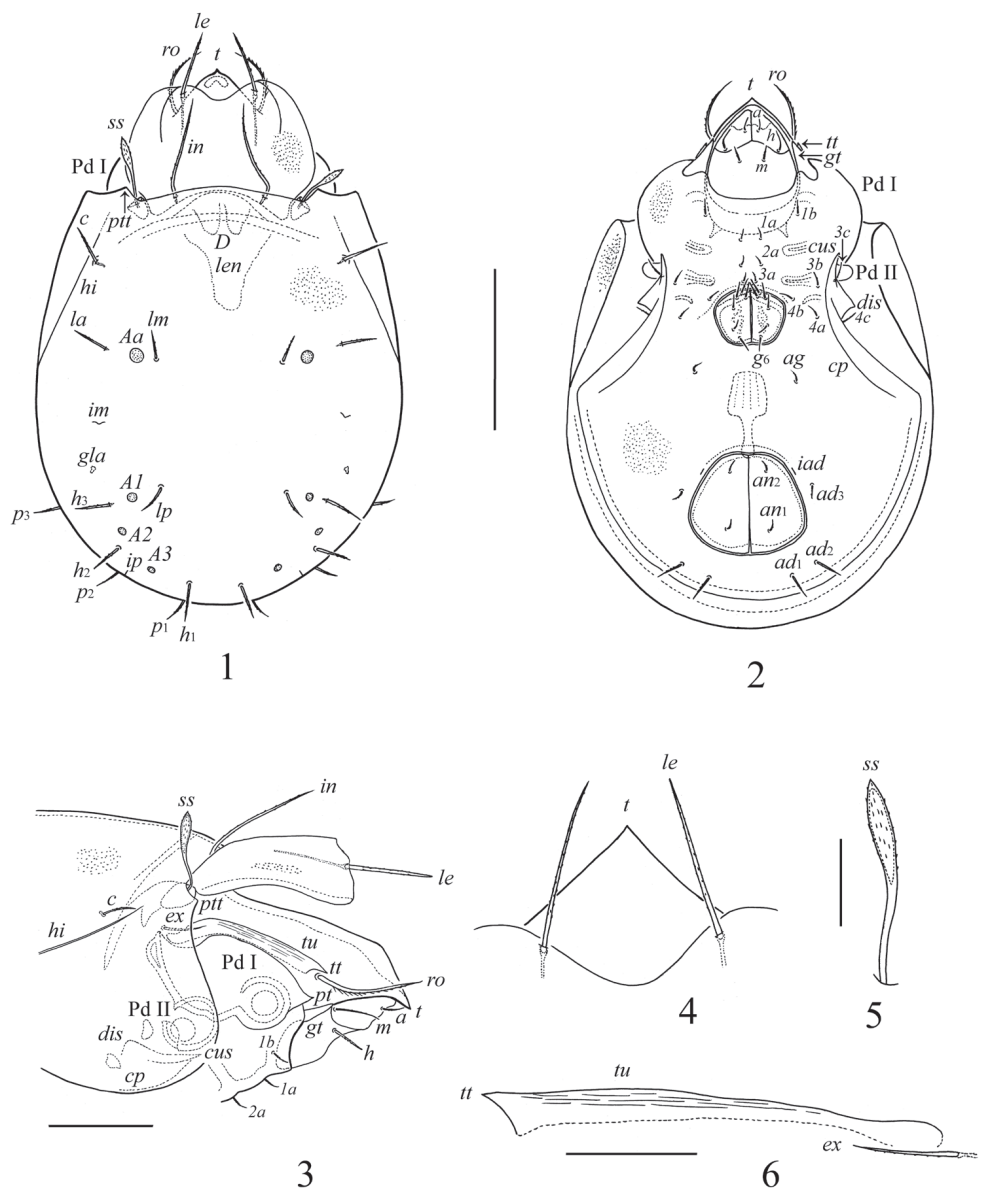
*Lepidozetes acutirostrum* sp. n., adult: **1** dorsal view **2** ventral view (legs not illustrated) **3** anterior part of body, lateral view (legs not illustrated)**4** rostrum, anterior margin of lamellae, lamellar setae, dorso-anterior view **5** sensillus **6** tutorium and exobothridial seta. Scale bar (**1, 2**) 200 μm, (**3**) 100 μm, (**4–6**) 40 μm.

**Figures 7–16.**
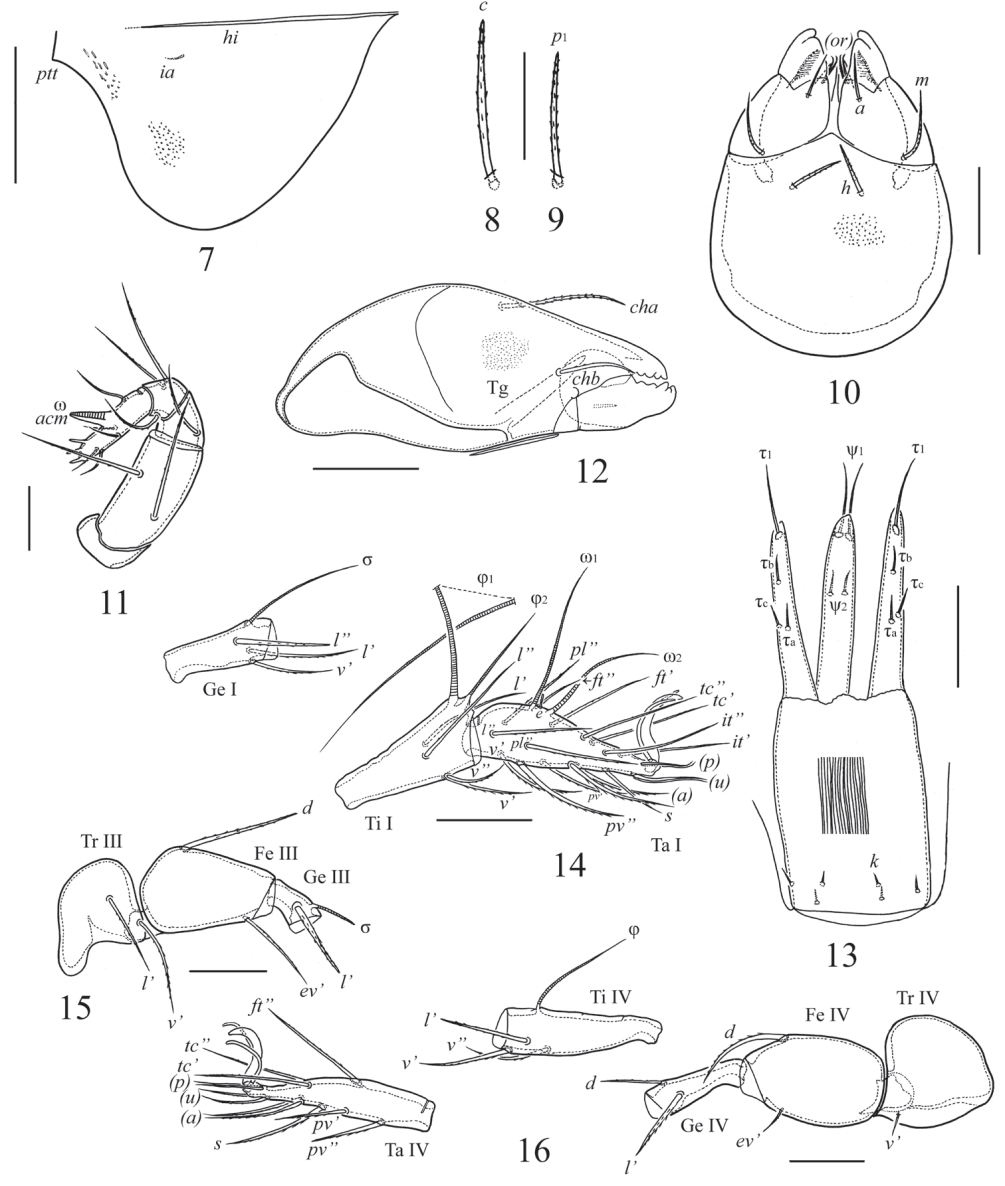
*Lepidozetes acutirostrum* sp. n., adult: **7** pteromorph, lateral view **8** notogastral seta *c* **9** notogastral seta *p*_1_ **10** subcapitulum, ventral view **11** palp **12** chelicera **13** ovipositor **14** genu (Ge), tibia (Ti) and tarsus (Ta) of leg I, right, antiaxial view **15** trochanter (Tr), femur (Fe) and genu of leg III, left, antiaxial view **16** leg IV, right, antiaxial view. Scale bar (**7**) 100 μm, (**8–10, 12, 14–16**) 40 μm, (**11**) 20 μm, (**13**) 50 μm.

#### Type deposition.

The holotype and one paratype are deposited in the collection of the Senckenberg Institution, Frankfurt, Germany; two paratypes are deposited in the collection of the Siberian Zoological Museum, Novosibirsk, Russia; three paratypes are deposited in the collection of the Tyumen State University Museum of Zoology, Tyumen, Russia.

#### Etymology.

The specific name “*acutirostrum*” refers to the pointed rostrum.

#### Comparison.

*Lepidozetes acutirostrum* sp. n. can be distinguished from all known species of the genus *Lepidozetes* using the key, which is presented below.

### Key to known species of the genus *Lepidozetes*

**Table:** 

1	Sensilli clavate, with head rounded distally; interlamellar setae clearly shorter than sensilli	2
–	Sensilli lanceolate, with head pointed distally or disk-like; interlamellar setae clearly longer than sensilli	3
2	Lamellae rounded anteriorly; leg tarsi with three claws; larger body size: 373–442 × 248–391	*Lepidozetes singularis* Berlese, 1910 (=*Lepidozetes conjunctus* Schweizer, 1922; =*Lepidozetes chernovi* Ryabinin, 1974) (see Berlese 1910; Schweizer 1922; Hammer 1952; Krivolutsky and Ryabinin 1974; Mahunka 1993; Bayartogtokh and Aoki 1999) (Distribution: Holarctic region)
–	Lamellae concave anteriorly; leg tarsi with one claw; smaller body size: 300 × 229	*Lepidozetes trifolius* Fujikawa, 1972 (see Fujikawa 1972) (Distribution: Holarctic region)
3	Lamellae rounded anteriorly; sensilli with disk-like head; body size: 400 × 300	*Lepidozetes latipilosus* Hammer, 1952 (see Hammer 1952) (Distribution: Holarctic region)
–	Lamellae concave anteriorly; sensilli with lanceolate head	4
4	Rostrum pointed; larger body size: 647–697 × 431–481	*Lepidozetes acutirostrum* sp. n. (Distribution: northern Nepal)
–	Rostrum rounded; smaller body size: 500–568 × 348–424	*Lepidozetes dashidorzsi* Balogh et Mahunka, 1965 (see Balogh and Mahunka 1965; Fujikawa 1972; Bayartogtokh and Aoki 1999) (Distribution: south-eastern Palearctic region)

### Scutozetes clavatosensillus

Ermilov, Martens & Tolstikov sp. n.

http://zoobank.org/1D0CFEF9-4751-4A75-A5C5-0E55D418468A

http://species-id.net/wiki/Scutozetes_clavatosensillus

Figs 17 –27

#### Diagnosis.

Body size 415–448 × 265–273. Rostrum broadly rounded. Lamellae not covering rostrum and lateral sides of prodorsum. Anterior margin of lamellae weakly concave medially. Interlamellar setae longer than rostral and lamellar setae. Sensilli clavate. Tutoria triangular distally, with two to five small teeth anteriorly. Anterior margins of pteromorphs triangular. Notogastral setae of medium size, weakly thickened, barbed. Genal teeth broadly triangular. Adanal setae *ad*_1_, *ad*_2_ longer than other anogenital setae.

#### Description.

*Measurements*. Body length 431 (holotype: female), 415–448 (five paratypes: three females and two males); body width 265 (holotype), 265–273 (five paratypes).

*Integument*. Body color light brown. Body surface microfoveolate (diameter of foveolae up to 1), but visible only under high magnification (× 1000) in dissected specimens. Dorsal sides of lamellae with longitudinal striae.

*Prodorsum*. Rostrum broadly rounded. Lamellae of medium size, not covering rostrum and lateral sides of prodorsum. Anterior margin of lamellae weakly concave medially. Rostral setae (57–65) setiform, ciliate, directed anterio-mediad, inserted laterally on prodorsum; their basal parts covered by the tutoria. Lamellar setae (41–49) straight, slightly barbed, directed forward, inserted dorso-anteriorly on lamellae. Interlamellar setae (73–82) setiform, slightly barbed, directed upwards and forwards, inserted on posterior part of lamellae; basal parts of these setae covered by the anterior margin of notogaster. Sensilli (45–53) clavate, with long stalk and oval head rounded or weakly truncated distally. Tutoria long, of medium width, triangular distally, with two to five small teeth anteriorly. Exobothridial setae (24) setiform, thin, slightly barbed, inserted dorso-posteriorly to tutoria.

*Notogaster*. Pteromorphs concave laterally. Anterior margins of pteromorphs triangular, longer than length of sensilli. Dorsophragmata located close to each other. Postero-median part of hinges distinct, anterior part unvisible. Lenticulus indistinctive. Four pairs of sacculli (*Sa*, *S1*, *S2*, *S3*) developed. Ten pairs of notogastral setae weakly thickened, barbed: setae *c* and *la* (both 28–32) longer than other setae (16–24). Lyrifissures *ia*, *im*, *ip*, *ih* and *ips* and opisthonotal gland openings located typically for the genus.

*Gnathosoma*. Subcapitulum longer than wide (110–114 × 86–90). Subcapitular setae *h* (16–20) thickened, straight, barbed; *a* (12–16) and *m* (24–28) little thinner, slightly barbed. Two pairs of adoral setae (10–12) setiform, hook-like distally, barbed. Palps (length 69–77) with setation 0–2–1–3–9(+ω). Solenidion weakly thickened, straight, blunt-ended, attached with eupathidium. Chelicerae (length 114) with two barbed setae; *cha* (36) longer than *chb* (24). Trägårdh’s organ long, conical.

*Lateral podosomal and epimeral regions*. Genal teeth broadly triangular. Pedotecta I of medium size, rounded anteriorly. Pedotecta II small, scale-like. Apodemes 1, 2, 3 and sejugal distinctly developed, not fused medially. Epimeral setal formula 3–1–3–3; setae (10–12) setiform, slightly barbed. Custodia with thin, free, blunted tips, directed anteriorly to the pedotecta II. Discidia pointed. Circumpedal carinae distinct.

*Anogenital region*. Six pairs of genital (8–12), one pair of aggenital (8–12), two pairs of anal (8–12) and three pairs of adanal (*ad*_1_, *ad*_2_, 14–16; *ad*_3_, 10–12) setae setiform, barbed. Lyrifissures *iad* located in paraanal position.

*Legs*. Similar to *Lepidozetes acutirostrum* sp. n.

**Figures 17–20.**
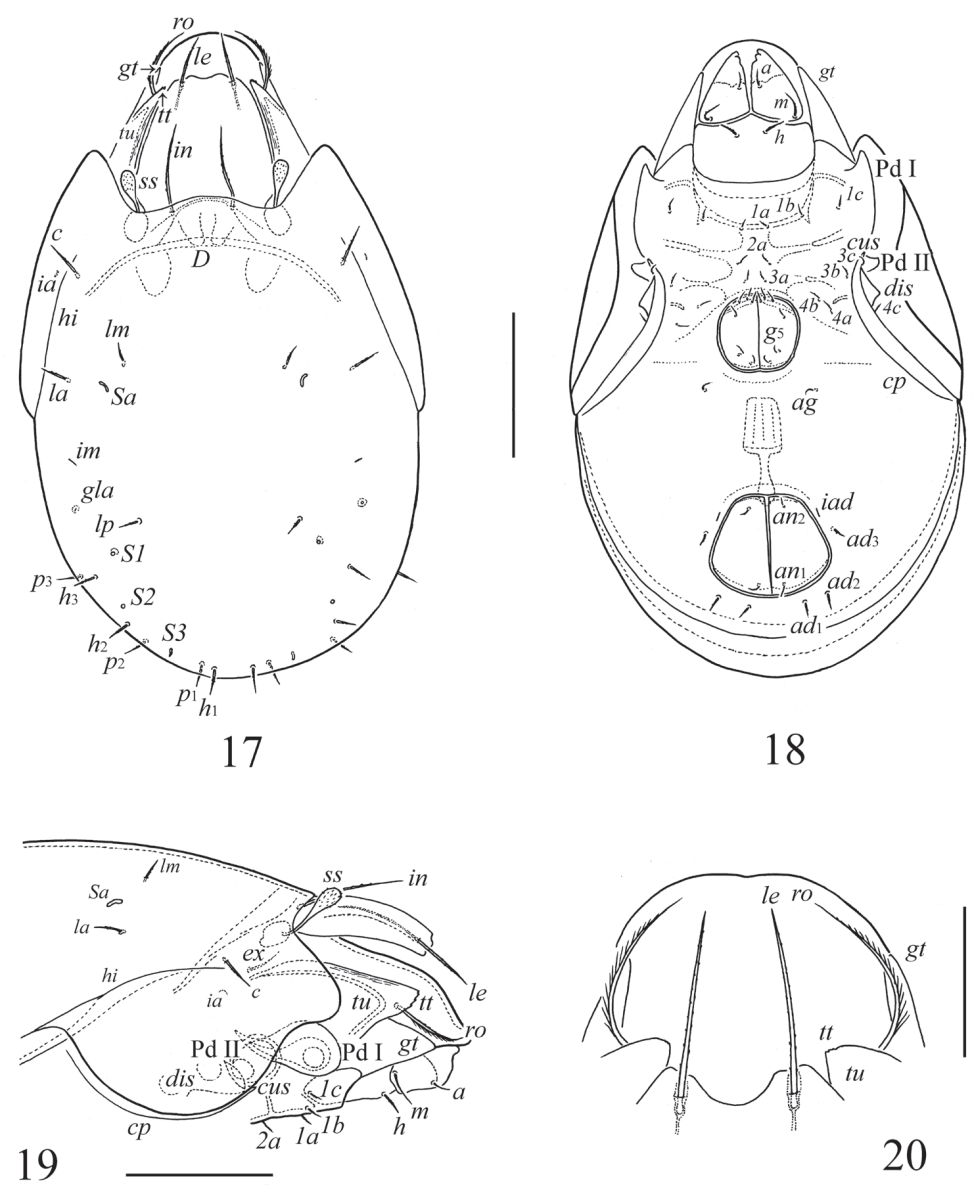
*Scutozetes clavatosensillus* sp. n., adult: **17** dorsal view **18** ventral view (legs not illustrated) **19** anterior part of body, lateral view (legs not illustrated)**20** rostrum, anterior margin of lamellae and tutoria, rostral and lamellar setae, dorso-anterior view. Scale bar (**17–19**) 100 μm, (**20**) 40 μm.

**Figures 21–27.**
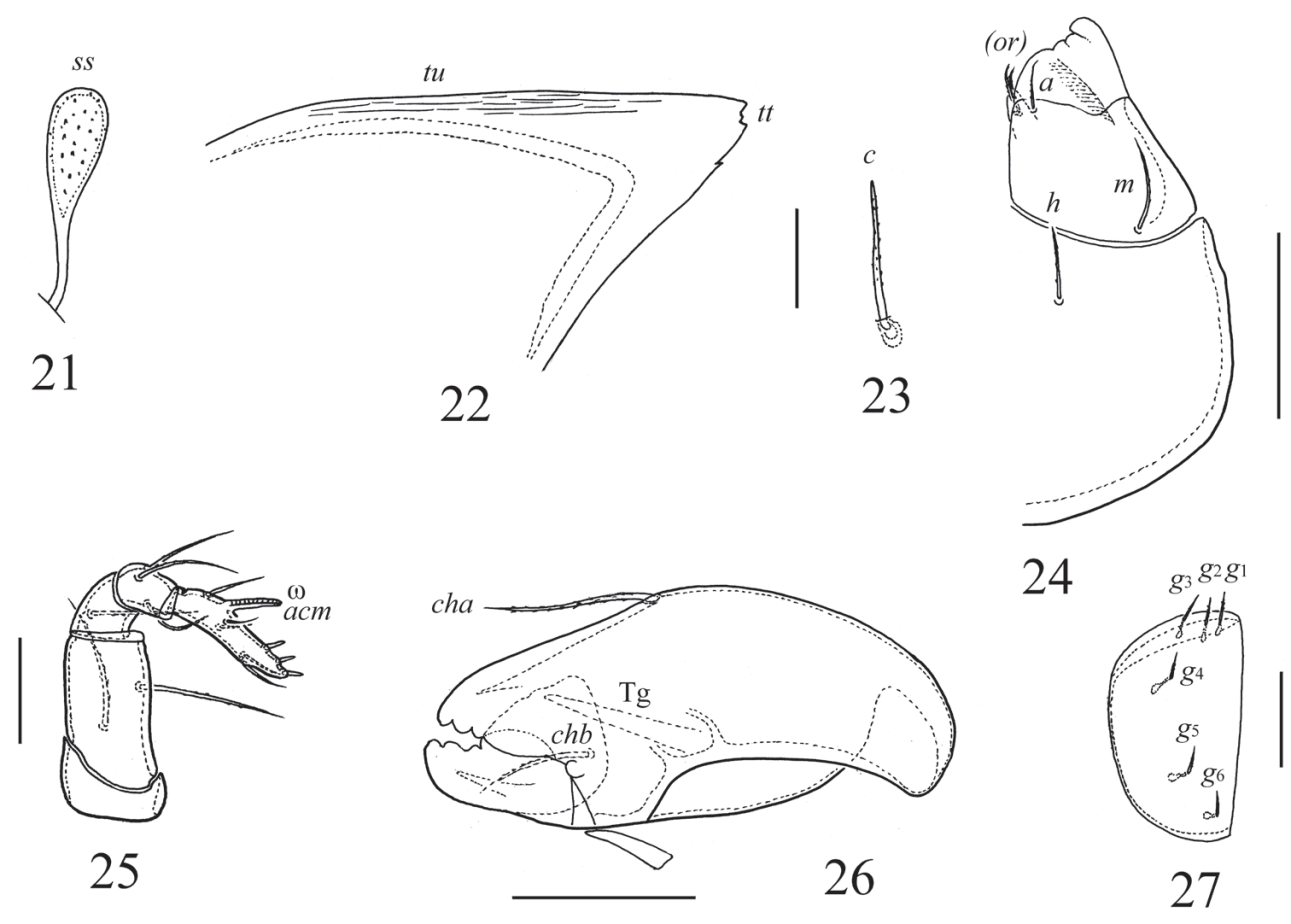
*Scutozetes clavatosensillus* sp. n., adult: **21** sensillus **22** tutorium **23** notogastral seta *c* **24** left half ofsubcapitulum, ventral view **25** palp **26** chelicera **27** right genital plate. Scale bar (**21–23, 25, 27**) 20 μm, (**24, 26**) 40 μm.

#### Type deposition.

The holotype and one paratype are deposited in the collection of the Senckenberg Institution, Frankfurt, Germany; two paratypes are deposited in the collection of the Siberian Zoological Museum, Novosibirsk, Russia; two paratypes are deposited in the collection of the Tyumen State University Museum of Zoology, Tyumen, Russia.

#### Etymology.

The specific name “*clavatosensillus*” refers to the clavate sensilli.

#### Comparison.

*Scutozetes clavatosensillus* sp. n. can be distinguished from all known species of the genus *Scutozetes* by the key, which is presented below.

### Key to known species of the genus *Scutozetes*

**Table:** 

1	Sensilli lanceolate, with head pointed distally; anterior margins of pteromorphs slightly projecting forward; body size: 437–484 × 320–390	*Scutozetes lanceolatus* Hammer, 1952 (see Hammer 1952; Fujikawa 1972; Mahunka 1993) (Distribution: Holarctic and Neotropical regions, Surinam)
–	Sensilli clavate, with head rounded distally; anterior margins of pteromorphs strongly projecting forward, triangular-form	2
2	Lamellae large, covering lateral side of prodorsum, broadly rounded anteriorly; interlamellar setae reach the insertions of lamellar setae; body length: 420	*Scutozetes ovalis* (Hammer, 1977) (see Hammer 1977) (Distribution: Pakistan, Korea)
–	Lamellae of medium size, not covering lateral side of prodorsum, concave anteriorly; interlamellar setae do not reach the insertions of lamellar setae; body size: 415–448 × 265–273	*Scutozetes clavatosensillus* sp. n. (Distribution: North Nepal)

## Supplementary Material

XML Treatment for Lepidozetes acutirostrum

XML Treatment for Scutozetes clavatosensillus

## Footnotes

^1^ Results of the Himalaya Expeditions of Dr. Jochen Martens.

^2^ Subías (2004, online version 2013) includes the genus *Onazetes* Bugrov, 1991 with the type species, *Onazetes umbellatus* Bugrov, 1991 (see Bugrov 1991), in *Lepidozetes*. However, *Onazetes umbellatus* has an additional pair of porose areas close to notogastral setae c (it absent in all *Lepidozetes* species) and five pairs of genital setae (six pairs in all *Lepidozetes*-species). Further research is needed to establish the taxonomical position of *Onazetes*.
